# Evaluation of Antioxidant Activity of Residue from Bioethanol Production Using Seaweed Biomass

**DOI:** 10.3390/md22080340

**Published:** 2024-07-26

**Authors:** In-Yung Sunwoo, Hyunjin Cho, Taeho Kim, Eun-Jeong Koh, Gwi-Taek Jeong

**Affiliations:** 1 Jeju Marine Research Center, Korea Institute of Ocean Science and Technology (KIOST), Jeju 63349, Republic of Korea; iysunwoo@kiost.ac.kr (I.-Y.S.); kt1024@kiost.ac.kr (T.K.); kej763@kiost.ac.kr (E.-J.K.); 2 Department of Biotechnology, Pukyong National University, Busan 48513, Republic of Korea; chjin0801@gmail.com

**Keywords:** seaweeds, ethanol production, fermentation, antioxidant activity

## Abstract

This study explores the potential of producing bioethanol from seaweed biomass and reusing the residues as antioxidant compounds. Various types of seaweed, including red (*Gelidium amansii*, *Gloiopeltis furcata*, *Pyropia tenera*), brown (*Saccharina japonica*, *Undaria pinnatifida*, *Ascophyllum nodosum*), and green species (*Ulva intestinalis*, *Ulva prolifera*, *Codium fragile*), were pretreated with dilute acid and enzymes and subsequently processed to produce bioethanol with *Saccharomyces cerevisiae* BY4741. Ethanol production followed the utilization of sugars, resulting in the highest yields from red algae > brown algae > green algae due to their high carbohydrate content. The residual biomass was extracted with water, ethanol, or methanol to evaluate its antioxidant activity. Among the nine seaweeds, the *A. nodosum* bioethanol residue extract (BRE) showed the highest antioxidant activity regarding the 2,2-diphenyl-1-picrylhydrazyl (DPPH) activity, ferric reducing antioxidant power (FRAP), and reactive oxygen species (ROS) inhibition of H_2_O_2_-treated RAW 264.7 cells. These by-products can be valorized, contributing to a more sustainable and economically viable biorefinery process. This dual approach not only enhances the utilization of marine resources but also supports the development of high-value bioproducts.

## 1. Introduction

Fossil fuels such as natural gas, oil, and coal are widely used around the world. However, due to industrialization and population growth, the use of fossil fuels is increasing, raising concerns about their depletion in the near future [1,2]. This has led to a growing demand for alternative energy sources and a decreasing dependence on fossil fuels [3]. Alternative energy sources include solar energy, wind power, hydroelectric power, and bioenergy [4]. Among these, bioenergy stands out as a prominent alternative to fossil fuels because it can be produced in many countries, is suitable for road transportation fuel, and contributes to reducing harmful environmental impacts such as air pollution and greenhouse gas emissions [5,6].

The demand for renewable energy has significantly increased globally, and the production of bioethanol from various sources has been extensively studied. Microalgae have been researched as feedstocks for third-generation bioethanol production due to their high carbohydrate content, ability to capture CO_2_ from the atmosphere, and advantages in food production [7]. Seaweeds have also gained considerable attention in recent years as an alternative renewable feedstock due to their high biomass yields, rapid growth rates, and the fact that they do not require arable land for cultivation [8].

Many production processes for seaweed chemicals have traditionally focused on single products such as alginate, carrageenan, or pigments, with the remaining seaweed often treated as waste. Moreover, many current seaweed research programs are focused on single-product goals, with much research emphasizing biofuels. As an alternative to single-product processes, the concept of the biorefinery has been explored to maximize the inherent value of all components present in the biomass [9]. Seaweed is an excellent feedstock for such biorefineries because it contains high-value components (e.g., specialty polysaccharides and bioactive molecules), as well as compounds considered platform chemicals for the bio-based economy, such as glucose [10].

Numerous seaweed biorefinery processes have already been investigated for the production of biofuels and commodity chemicals. For instance, in the red seaweed species *Gracilariopsis longissima* (formerly *Gracilaria verrucose*), after extracting agar, the residual was converted to bioethanol, achieving an ethanol yield of 0.43 g/g of sugar [11]. Integrated processes have also been developed for green seaweed *Ulva* spp., sequentially recovering economically important components such as mineral-rich liquid extracts, lipids, ulvan, and cellulose [12]. Various biorefinery processes using brown seaweeds such as *Saccharina latissima* and *Ascophyllum nodosum* have been studied for mannitol separation and the production of fucoidan, alginate, sugars, and biochar [13]. Additionally, biorefinery scenarios with *Laminaria digitata* have been explored for bioethanol and succinic acid production, with the remaining residues analyzed as potential feedstocks for biogas production, biodiesel, and feed supplements [14]. Additionally, various nutrients, pigments, and even sea salt can be extracted from seaweed, enhancing the potential value of biorefinery processes [15].

Recently, researchers have focused on evaluating extracts generated from the hydrolysates of various pretreatment processes in the context of integrated biorefineries. However, studies on the antioxidant capacity of extracts generated from post-ethanol distillation residues are limited. The capacity varies depending on various factors, such as the raw materials, pretreatment, and extraction method. In this study, bioethanol was produced from hydrolysates obtained after the dilute acid pretreatment of seaweed biomass and pretreated biomass. The hydrolysate residues were reused, and their antioxidant activity was evaluated after extraction with water, ethanol, and methanol.

## 2. Results 

### 2.1. Characterization of the Biomass

The compositions and origins of the seaweeds used in this study are shown in Table 1. The average carbohydrate content was the highest in red algae at 63%, followed by brown algae at 59.63% and green algae at 37.05%. The results suggest that higher carbohydrate content can lead to higher bioethanol production, as carbohydrates are the only substrates that can support fermentation for bioethanol production. Additionally, the storage carbohydrates and non-fermentable sugars in each type of seaweed are known to have beneficial effects, such as antioxidant and anti-inflammatory activity [16].

The significance of the findings lies in the potential of red and brown algae as superior candidates for bioethanol production due to their high carbohydrate content. Carbohydrates, primarily in the form of polysaccharides such as cellulose and starch, are essential for the fermentation process involved in bioethanol production [17]. Therefore, seaweeds with higher carbohydrate content can theoretically yield more bioethanol, making them more efficient for industrial biofuel applications. Moreover, beneficial bioactive compounds derived from storage carbohydrates and non-fermentable sugars can add value to the by-products of bioethanol production, promoting the use of seaweed in the nutraceutical and pharmaceutical industries [18].

The average protein content of the green algae, red algae, and brown algae was 23.56%, 21.84%, and 19.23%, respectively. Seaweed proteins can mainly be divided into phycoerythrin, glycoproteins, enzymes, phycobiliproteins, and mycosporine-like amino acids. Glycoproteins in the cell wall are the main type of protein in most seaweeds and are involved in physiological functions [19].

The protein content of seaweeds not only contributes to their nutritional value but also determines their industrial utility. Proteins such as phycoerythrin and phycobiliproteins have significant commercial value due to their applications in the food, cosmetics, and biomedical fields. For example, phycoerythrin can be used as a natural colorant and in fluorescent markers for biomedical research [20]. Glycoproteins, which constitute a substantial portion of seaweed proteins, are involved in various physiological processes and can be harnessed for their functional properties in the development of therapeutic agents.

The lipid content of brown algae was the highest on average at 3.1%, followed by green algae at 1.60% and red algae at 0.80%. In comparison with other marine organisms, seaweeds have lower lipid content, constituting only 4.5–6.7% of the dry weight [21]. Seaweeds are rich in bioactive lipids such as phospholipids, glycolipids, and sterols (e.g., fucosterol), which are reported to have various physiological properties, including anti-cancer, antioxidant, antiviral, anti-diabetes, and hypocholesterolemic activity [22].

Although the lipid content of seaweeds is relatively low compared with that of other marine organisms, the presence of bioactive lipids is noteworthy. These lipids, particularly phospholipids and sterols, have significant health benefits, making them valuable for the development of functional foods and pharmaceuticals. For example, fucosterol has been extensively studied for its anti-inflammatory and cholesterol-lowering effects [23]. The identification of these bioactive compounds in seaweeds demonstrates their potential as a source of nutraceutical components.

The composition of seaweed is highly sensitive to seasonal, environmental, geographical, and other factors, making it difficult to generalize the algal composition [24]. However, the chemical composition of seaweed can affect its industrial applications in biofuel production and its antioxidant efficacy. Therefore, the selection of the biomass is considered the most important step in the overall process. In this study, nine types of seaweed were selected, and experiments were conducted to compare their bioethanol production and antioxidant capabilities using the same process.

**Table 1:** Composition of seaweeds.

Species	Seaweed	Composition			
		Carbohydrate	Crude Protein	Crude Lipid	Crude Ash
Red seaweed	*Gelidium amansii* [25]	74.40	7.27	0.03	18.30
	*Gloiopeltis furcata* [26]	62.56	24.47	0.23	12.74
	*Pyropia tenera*	52.04	33.77	2.15	12.04
Brown seaweed	*Ascophyllum nodosum* [27]	69.70	23.30	4.20	2.80
	*Sacchrina japonica* [28]	66.00	10.60	1.60	21.80
	*Undaria pinnatifida*	43.20	23.80	3.50	29.50
Green seaweed	*Codium fragile*	34.24	10.64	2.23	52.89
	*Ulva* *intestinalis*	31.60	29.20	1.80	37.40
	*Ulva prolifera*	45.30	30.84	0.78	23.08

### 2.2. Bioethanol Production from Nine Seaweeds

Experiments were conducted under identical conditions to produce fermentable sugars from red algae, brown algae, and green algae for bioethanol production with thermal acid hydrolysis and enzymatic saccharification. As shown in Table 2, monosaccharides were first hydrolyzed from carbohydrates by thermal acid hydrolysis, achieving 32.45–40.00% conversion, and further enzymatic saccharification increased this to 78.76–83.12%, demonstrating significant monosaccharide yields proportional to the carbohydrate content. Following pretreatment and enzymatic saccharification, red algae yielded an average of 37.87 g/L of monosaccharides, brown algae produced 36.584 g/L, and green algae provided 22.48 g/L. In terms of fermentable sugars, red algae yielded glucose and galactose, brown algae provided glucose and mannitol, while green algae contributed glucose. Thus, it was observed that red algae provided the highest, followed by brown algae and green algae.

Generally, physicochemical and biological methods are used to break down polysaccharides into monosaccharides. Acid hydrolysis is the most common and preferred method for biomass pretreatment because the cost of acid is cheaper and it can be performed faster than other processes [29]. Additionally, studies have shown that a two-step process can at least double the yield of fermentable sugars [30]. Recent studies have further highlighted the importance of optimizing both the pretreatment and hydrolysis methods to enhance the monosaccharide yields from seaweed. To secure fermentable sugars, the saccharification method involving simultaneous and consolidated saccharification and enzymatic hydrolysis has been reported to increase the bioethanol yield by 20% [31]. This method was effective in most cases where the seaweed’s polysaccharide content was low or uniform.

**Figure 1:**
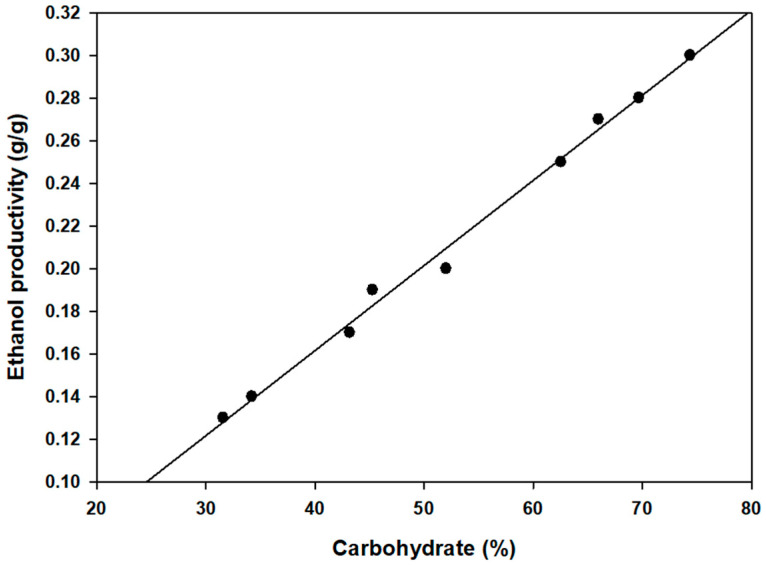
Relation of ethanol productivity and carbohydrate concentration.

Fermentation was conducted using *S. cerevisiae* BY4741, capable of consuming glucose, galactose, and mannitol. Ethanol production was assessed after 144 h of fermentation. Ethanol production followed the utilization of sugars, resulting in the highest yields from red algae > brown algae > green algae. The ethanol production results are summarized in Table 2. Among the red algae, *G. amansii* was the top ethanol producer. For brown algae, *A. nodosum* and *S. japonica* surpassed *G. furcata* in ethanol production. Green algae exhibited the lowest ethanol production, with *U. prolifera* showing the highest yields. This trend correlates with the carbohydrate content, emphasizing the necessity of high-carbohydrate biomass for efficient bioethanol production (Figure 1).

The productivity of ethanol in the previous study showed a wide range for the same seaweeds used in this study [32]; *G. amansii*, 0.38–0.47 g/g, *S. japonica* 0.16–0.45 g/g, *U. pinnatifida* 0.14 g/g, *U. intestinalis* 0.21 g/g. In this study, the ethanol production ratio per gram of biomass ranged from 0.12 to 0.30. These indicate that the processes related to bioethanol production from seaweed, including the selection of high biomass content, thermal acid hydrolysis, enzymatic hydrolysis, and fermentation, require techno-economic upgrades to achieve higher yields.

### 2.3. Antioxidant Activity of Bioethanol Residue Extracts (BRE)

The extracts were obtained using water, ethanol, or methanol as solvents. For *G. furcata*, water extraction was not possible due to its high water absorption rate. The high water absorption rate of *G. furcata* suggests that the seaweed’s polysaccharide content, such as agar or carrageenan, may lead to strong hydrophilic properties. Due to specific properties such as gelation and gloss film formation, *G. furcata* is typically used for three purposes, i.e., facing, consolidation, and thickeners in conservation [33].

**Figure 2:**
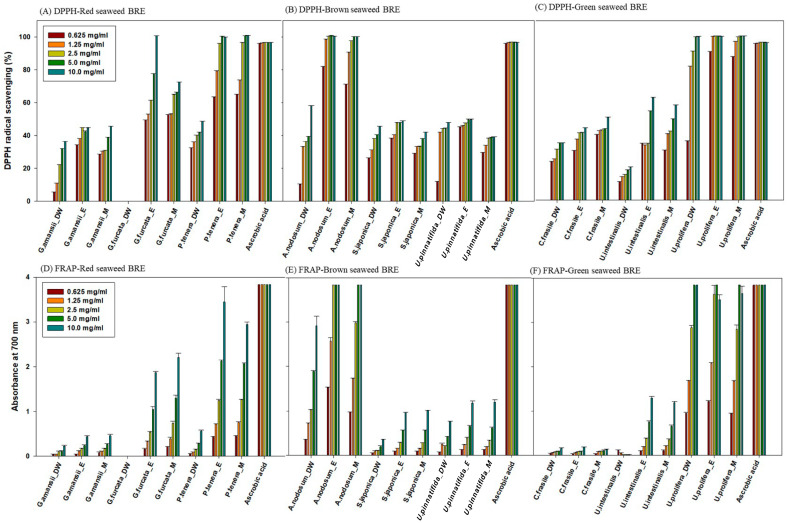
Antioxidant activity of BRE tested with DPPH with (**A**) red seaweed, (**B**) brown seaweed, (**C**) green seaweed and with FRAP with (**D**) red seaweed, (**E**) brown seaweed, and (**F**) green seaweed.

The BRE used in the experiments were dissolved in DMSO to achieve a final concentration of 0.625–10 mg/mL after removing the organic solvents from each extract. The results from both the DPPH and FRAP assays revealed significant variations in the antioxidant activity among the different seaweed species and extraction solvents (Figure 2). The patterns observed suggest that specific combinations of seaweed species and solvents can maximize the antioxidant potential.

For red algae, the ethanol and methanol BRE of *P. tenera* exhibited the highest antioxidant activity, starting from a concentration of 2.5 mg/mL. Therefore, the *P. tenera* BRE could be a particularly potent source of antioxidants when extracted with these solvents. Additionally, the ethanol extract of the *G. furcata* BRE demonstrated high efficacy at 10 mg/mL, indicating that, despite its efficacy, a higher concentration is required to achieve significant antioxidant effects compared with those of the *P. tenera* BRE. The other red algal species showed antioxidant activity below 80%, suggesting a relatively lower level or efficacy of antioxidant compounds.

The FRAP assay results were in agreement with the DPPH assay results, with the ethanol and methanol extracts of the *P. tenera* BRE showing the highest antioxidant activity, followed by the BRE of *G. furcata*. The consistent observations across the different assays demonstrate the robustness of the results regarding the antioxidant capacity of these extracts.

In the case of brown algae, the ethanol and methanol extracts of the *A. nodosum* BRE exhibited the highest antioxidant activity. Therefore, the antioxidant compounds of the *A. nodosum* BRE may be more effectively extracted using these solvents. Although to a lesser degree, the water extract of the *A. nodosum* BRE also showed significant antioxidant activity, indicating the presence of water-soluble antioxidant compounds; however, they may be present at lower concentrations or with lower potency compared with those extracted with ethanol and methanol.

For green algae, the ethanol extract of the *U. prolifera* BRE showed the highest antioxidant activity. Both the water and methanol extracts of the *U. prolifera* BRE also exhibited high antioxidant activity at higher concentrations, suggesting that a wide range of antioxidant compounds may be effective with different solvent systems. The findings highlight the *U. prolifera* BRE as a versatile source of antioxidants, with compounds that may be efficiently extracted using both polar and non-polar solvents.

For a clearer comparison between the seaweeds and extraction solvents, the IC50 values of DPPH are presented in Table 3. Overall, the findings indicate that ethanol and methanol may be more effective than water for the extraction of antioxidant compounds from seaweeds. This may be attributed to their ability to solubilize a broader spectrum of phytochemicals [34]. Notably, in the BRE from the three species of seaweed (*P. tenera*, *A. nodosum*, and *U. prolifera*), the IC50 values for ethanol and methanol were found to be below 2.00 mg/mL. Therefore, the selection of appropriate solvent systems, particularly ethanol and methanol, is crucial in maximizing the extraction of antioxidant compounds from seaweeds.

Previous studies have demonstrated strong antioxidant activity in ethanol extracts of *G. amansii* (IC50 0.17 mg/mL) [35]. However, this study found that the biomass used for bioethanol production exhibited reduced antioxidant activity, suggesting that the antioxidant components of red algae are diminished during bioethanol production. It was reported that extraction at high temperatures (e.g., 100 °C) could destroy some bioactive compounds and lead to a lack of antioxidant activity [36]. Some bioactive compounds vital for antioxidant activity may be destroyed during high-temperature pretreatment, leading to reduced antioxidant activity in the other seaweed extracts.

However, considering that significant activity was observed only in the BRE of *P. tenera*, *A. nodosum*, and *U. prolifera*, this indicates that seaweeds with retained antioxidant activity could be used to recycle the slurry remaining after bioethanol production.

Furthermore, the consistency between the DPPH and FRAP assay results suggests that the outcomes are reliable and reproducible across different methodologies [37]. The findings have significant implications for the potential use of seaweed extracts in various industries, including food, cosmetics, and pharmaceuticals, where antioxidants play a crucial role in health and preservation.

### 2.4. Cell Viability in the Presence of BRE

**Figure 3:**
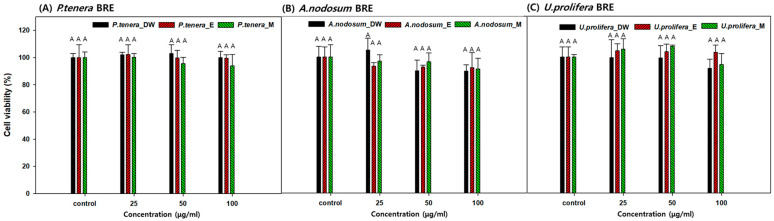
Effects of BRE in RAW 264.7 cells treated with (**A**) red seaweed, (**B**) brown seaweed, and (**C**) green seaweed. Cell viability was assessed using the MTT method. Control: not treated with any extract. Data are presented as the means of the percentage of the control cells ± the SD of triplicate data. Means with letter A above the bars were significantly different in Duncan’s multiple range test at *p* < 0.05.

**Figure 4:**
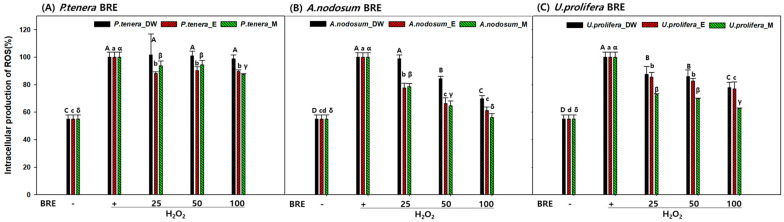
Effects of BRE on RAW 264.7 cells treated with H_2_O_2_ and (**A**) red seaweed, (**B**) brown seaweed, and (**C**) green seaweed. ROS production was evaluated using the DCF-DA dye. Control: not treated with any extract or H_2_O_2_. Data are presented as the means of the percentage of the control cells ± the SD of triplicate data. Means with different letters (A–D, a–d, α–δ) above the bars were significantly different in Duncan’s multiple range test at *p* < 0.05.

The toxicity and antioxidant capacity of red algae (*P. tenera* BRE), brown algae (*A. nodosum* BRE), and green algae (*U. prolifera* BRE) were evaluated, as shown in Figure 3 and Figure 4. No toxicity was observed in any of the samples. The absence of cytotoxicity in all BRE at concentrations of 25, 50, and 100 µg/mL indicates that these extracts may be safe for use in biological applications, such as in food supplements or pharmaceuticals [38].

In the ROS inhibition experiment, the *A. nodosum* BRE exhibited the highest inhibitory activity, followed by the *U. prolifera* BRE. The *P. tenera* BRE showed significant ROS inhibition activity, but to a lesser extent compared to the BRE of *A. nodosum* and *U. prolifera*.

Among the seaweeds tested, the brown algae *A. nodosum* BRE demonstrated the strongest inhibition of intracellular ROS production compared to the *P. tenera* and *U. prolifera* BRE. The results were consistent between the DPPH and FRAP assays, indicating the presence of potent antioxidant compounds in the *A. nodosum* BRE, likely comprising known antioxidant polysaccharides. The high efficacy of *A. nodosum* observed in this study is consistent with the findings of previous studies showing significant antioxidant activity under specific pretreatment conditions [39]. Moreover, the effectiveness of the antioxidant polysaccharides in *A. nodosum* has been reported. It is known that fucoidan can generally be extracted using a large amount of an aqueous or acidic solution at a temperature ranging from room temperature to 100 °C for several hours. Previous studies have reported high efficacy following pretreatment at 83 °C for 4 h compared with low-temperature treatment, which is in agreement with the results of this study [40].

Interestingly, the red algae *P. tenera*, known for its antioxidant properties, showed lower intracellular ROS inhibition in this study. The antioxidant properties of the *P. tenera* BRE may be affected by the preprocessing and fermentation stages. Previous studies have reported similar results. *P. tenera* extracted at 100 °C exhibited low levels of flavonoid content, DPPH, ABTS, and phenol content, indicating its low antioxidant activity [36]. This outcome might be attributed to the loss of antioxidant compounds during preprocessing and fermentation, processes known to affect the stability and efficacy of bioactive compounds.

The green algae *U. prolifera* BRE also exhibited substantial ROS inhibition, particularly in its ethanol extract, followed by its water and methanol extracts at higher concentrations. The variability in the antioxidant activity across different solvents suggests that *U. prolifera* may contain a wide range of antioxidant compounds with varying solubility. The loss of antioxidant activity in *U. prolifera* extracts might be attributed to the degradation of light-sensitive pigments [41].

The findings suggest that *A. nodosum* and *U. prolifera* hold significant potential as sources of natural antioxidants. However, a comparison of their bioethanol production capabilities revealed that the productivity of *A. nodosum* was two-fold higher than that of *U. prolifera*. Therefore, *A. nodosum* should be explored further for applications in bioenergy fuel production and dietary supplements, leveraging its robust antioxidant properties. The insights gained from this study could guide the development of effective extraction and processing methods to maximize the yields and stability of antioxidant compounds derived from seaweeds.

Given the variability in seaweed’s composition due to external factors, it is crucial to carefully select and characterize the biomass for specific industrial applications. By comparing different types of seaweed under controlled conditions, this study aimed to identify the most promising candidates for bioethanol production and antioxidant applications. This approach not only optimizes the use of seaweeds in biofuel production but also facilitates the development of high-value bioproducts, thereby contributing to the sustainable utilization of marine resources.

## 3. Materials and Methods

### 3.1. Biomass

Nine biomass types were used in this study. *Gelidium amansii* (Jindo, Korea), *Gloiopeltis furcata* (Jeju, Korea) and *Pyropia tenera* (formerly Porphyra tenera) (Karimunjawa, Indonesia) for red seaweed; *Saccharina japonica* (Busan, Korea), *Undaria pinnatifida* (Busan, Korea), and *Ascophyllum nodosum* (Nova Scotia, Canada) for brown seaweed; and *Ulva intestinalis* (Wando, Korea), *Ulva prolifera* (Jeju, Korea), and *Codium fragile* (Wando, Korea) for green seaweed were used. The seaweeds were ground in a blender (Shinil, Seoul, Korea) and sieved with a 200-mesh sieve (Chunggye, Seoul, Korea). The seaweed powder was used for the experiments. The composition of the seaweeds was analyzed using the American Organization of Analytical Chemists (AOAC) methods [37] at the Feed and Foods Nutrition Research Center at Pukyong National University in Busan, Korea.

### 3.2. Ethanol Production

Dried powdered seaweed (8%, *w*/*v*) was treated with 1% (*v*/*v*) H_2_SO_4_ solution and autoclaved at 121 °C for 30 min. The resulting hydrolysate was neutralized to pH 6.0 using 10 N NaOH. Subsequently, an enzymatic saccharification process was conducted by mixing 8 units/mL of Celluclast 1.5 L (8.4 EGU/mL; Novozymes, Bagsvaerd, Denmark), Viscozyme L (1.2 FBG/mL; Novozymes, Bagsvaerd, Denmark), and Termamyl (120 KNU-T/g; Novozymes, Bagsvaerd, Denmark) and incubating the mixture at 45 °C and 150 rpm for 12 h.

*Saccharomyces cerevisiae* BY4741 was obtained from Soo-rin Kim’s lab at Kyungpook National University. The cell was cultured in YPD medium (10 g/L yeast extract, 20 g/L peptone, and 20 g/L D-glucose). The seed was incubated with agitation at 150 rpm at 30 °C for 24 h.

Fermentation was conducted in an Erlenmeyer flask containing 100 mL of the seaweed hydrolysate inoculated with *S. cerevisiae* BY4741 at a cell density of 0.35 g dcw/L. The fermentation process was maintained at 30 °C and 200 rpm for 5 days, with periodic sampling for analysis. The ethanol production yield (Y_EtOH_) was determined using the following formula:

Y_EtOH_ (g/g) = EtOH_MAX_/ΔS_PS_ (1)

where Y_EtOH_ represents the ethanol production yield (g/g), EtOH_MAX_ is the peak ethanol concentration achieved during fermentation (g/L), and ΔS_PS_ is the total increase in fermentable monosaccharide content (g/L) following pretreatment and saccharification. The theoretical maximum Y_EtOH_ is 0.51 based on the conversion of 1 mol of glucose to 2 mol of ethanol.

### 3.3. Seaweed Extraction

The ethanol production residue (10 g) was extracted with 100 mL of 80% (*v*/*v*) ethanol or 80% (*v*/*v*) methanol or with distilled water for 72 h at room temperature in a shaking incubator at 120 rpm. Then, the sample was centrifuged at 13,000× *g* for 20 min at 4 °C and filtered with Whatman filter paper no. 1 (Sigma-Aldrich, St. Louis, MO, USA). The supernatant was collected, and the slurry was re-extracted under the same conditions. This procedure was repeated 3 times. The supernatant was evaporated with a rotary evaporator under a vacuum at 40 °C to obtain the dried extract. The extract was freeze-dried, and the extraction yield was calculated as follows:

Yield_Extraction_ (%) = (W_1_/W_2_) × 100 (2)

where W_1_ is the weight of the dried extract and W_2_ is the weight of the freeze-dried sample. Freeze-dried extracts were stored at −20 °C and were dissolved in dimethyl sulfoxide (DMSO) for further analysis.

### 3.4. Antioxidant Analyses

#### 3.4.1. 2,2-Diphenyl-1-picrylhydrazyl (DPPH) Assay

Each extract was dissolved in DMSO and adjusted to a concentration of 0.05–10 mg/mL for use in the experiment. The DPPH solution was prepared by dissolving 3 mg of DPPH in 15 mL of ethanol, followed by mixing 1.5 mL of this solution with 3 mL of ethanol and 0.5 mL of DMSO. A sample volume of 50 μL was added to the DPPH solution and allowed to react at room temperature for 10 min. After the reaction, the absorbance was measured at 517 nm using a spectrophotometer. As a control, 50 μL of DMSO was used instead of the sample, and the absorbance value obtained from this reaction was determined. The DPPH radical scavenging activity was calculated using the following equation:

DPPH radical scavenging activity (%) = (1 − A/B) × 100(3)

where A is the absorbance of the sample and B is the absorbance of the control.

#### 3.4.2. Ferric Reducing Antioxidant Power (FRAP) Assay

For this experiment, 0.2 mL of an appropriately diluted sample was mixed with 200 mM sodium phosphate (pH 6.6) and 0.2 mL of 1% potassium ferricyanide. This mixture was then incubated at 50 °C for 20 min. After incubation, 0.2 mL of 10% trichloroacetic acid was added, and the mixture was centrifuged at 10,000 rpm for 10 min. To 0.5 mL of the supernatant, 0.5 mL of 0.1% ferric chloride was added, and the absorbance was measured at 700 nm using a spectrophotometer. Ascorbic acid was used as a control.

### 3.5. Cytotoxicity and Antioxidant Evaluation

#### 3.5.1. Cell Culture

RAW 264.7 cells were purchased from the Korean Cell Line Bank (Seoul, Korea) and cultured under controlled conditions at 37 °C with 5% CO_2_. RAW 264.7 macrophages were cultured in DMEM (Gibco, Grand Island, NY, USA) supplemented with 100 U/mL penicillin/streptomycin (Gibco) and 10% fetal bovine serum (FBS) (Gibco).

#### 3.5.2. Cell Viability Assay

The cytotoxicity levels of the extracts on culture cells were measured using the 3-(4,5-dimethylthiazol-22-yl)-2,5-diphenyltetrazolium bromide (MTT) assay [42]. RAW 264.7 cells (1 × 10^5^ cells/well) were dispensed into 96-well plates. On the next day, the medium was removed, and the cells were treated with water, ethanol, and methanol extracts from three seaweed species with the highest antioxidant activity at concentrations of 0, 50, and 100 µg/mL for 24 h. After incubation, 100 µL MTT was added and the mixture was incubated for 3 h. Then, 100 uL of DMSO was added to solubilize the formed formazan crystals, and the amount of formazan crystals was determined by measuring the OD at 570 nm using a microplate spectrophotometer.

#### 3.5.3. Determination of Intracellular ROS Scavenging Activity

To measure the generation of intracellular reactive oxygen species in response to oxidative stress, a dichlorofluorescin diacetate (DCFDA) assay was performed. RAW 264.7 cells (1 × 10^5^ cells/well) were dispensed into 96-well plates. On the next day, the cells were treated with water, ethanol, and methanol extracts from three seaweed species with the highest antioxidant activity at concentrations of 0, 50, and 100 µg/mL for 2 h, followed by treatment with 400 µM hydrogen peroxide for 30 min. Subsequently, 10 μM DCFDA was added, and the cells were incubated at 37 °C for 30 min. Intracellular fluorescence was measured using a fluorescence microplate reader with excitation at 485 nm and emission at 525 nm.

### 3.6. Analysis

Cell growth was assessed by measuring the optical density at 600 nm (OD_600_) using a UV spectrophotometer (Amersham Biosciences Ultrospec 6300 Pro; Biochrom, Cambridge, UK). The OD_600_ values were then converted into the dry cell weight (DCW) using a calibration curve correlating the DCW to the OD_600_ [43]. A pH meter (CH-8603; Mettler-Toledo AG, Schwerzenbach, Switzerland) was used to determine the pH levels.

The concentrations of glucose, xylose, 5-HMF, formic acid, levulinic acid, and ethanol were quantified using an HPLC instrument (1100 series; Agilent Technologies, Santa Clara, CA, USA) equipped with a refractive index detector. An Aminex HPX-87H column (300 mm × 7.8 mm; Bio-Rad, Hercules, CA, USA) was used for chromatographic separation. The eluent used was 5 mmol/L H_2_SO_4_, which had been filtered and degassed, flowing at a rate of 0.6 mL/min, and the analysis was performed at a temperature of 65 °C [44].

### 3.7. Statistical Analysis

All experiments were repeated three times per sample. The statistical significance of the experimental results was analyzed by one-way ANOVA, and Duncan’s multiple range test was performed at a significance level of *p* < 0.05. Statistical analysis was performed using the SAS 9.3 software (SAS Institute Inc., Cary, NC, USA).

## 4. Conclusions

This research demonstrates the feasibility of producing bioethanol from seaweed biomass, highlighting the potential of various red, brown, and green seaweed species as sustainable feedstocks. The study also shows that *A. nodosum* residues from the bioethanol production process possess significant antioxidant properties, which can be harnessed to create high-value bioproducts. This integrated approach of bioethanol production and residue valorization supports the development of a sustainable biorefinery model that maximizes the use of all components in the seaweed biomass. By optimizing the use of marine resources, this research contributes to the reduction of the dependence on fossil fuels and promotes environmental sustainability. Future work should focus on refining the extraction processes and scaling up production to fully realize the potential of seaweed-based biorefineries.

## Figures and Tables

**Table 2:** Results of ethanol production with nine seaweeds by pretreatment and fermentation.

Species	Seaweed	Efficiency of Pretreatment (%)	Initial Monosaccharides (g/L)			Ethanol (g/L)	Productivity (g EtOH/ g Biomass)
			Glucose	Galactose	Mannitol		
Red seaweed	*G. amansii*	82.19	27.11	21.81	-	23.97	0.30
	*G. furcata*	81.12	22.43	18.17	-	19.89	0.25
	*P. tenera*	78.76	20.23	12.56	-	16.07	0.20
Brown seaweed	*A. nodosum*	81.12	31.12	-	14.11	22.16	0.28
	*S. japonica*	82.45	32.12	-	11.41	21.33	0.27
	*U. pinnatifida*	79.12	21.12	-	6.22	13.40	0.17
Green seaweed	*C. fragile*	80.12	20.00	-	-	11.19	0.14
	*U* *. intestinalis*	79.45	18.90	-	-	10.24	0.13
	*U. prolifera*	83.12	28.53	-	-	15.36	0.19

**Table 3:** IC50 DPPH scavenging activity of different seaweed extracts.

DPPH IC50 (mg/mL)	Red Seaweed			Brown Seaweed			Green Seaweed		
	*G. amansii*	*G. furcata*	*P. tenera*	*A. nodosum*	*S. japonica*	*U. pinnatifida*	*C. fragile*	*U. intestinalis*	*U. prolifera*
H_2_O	10.62	-	5.69	6.41	6.51	5.47	7.96	17.07	2.07
Ethanol	5.14	2.55	1.79	1.80	4.69	4.21	5.62	5.10	1.85
Methanol	6.97	3.07	1.78	1.77	6.63	6.02	4.79	5.06	1.81

## Data Availability

The data presented in this study are available on request from the corresponding author.
